# The Position of War Consultants: The Government's New Policy

**Published:** 1915-07-10

**Authors:** 


### THE POSITION OF WAR CONSULTANTS.

### The Government's New Policy.

Sir William Osler recently called attention to the discovery that the war consultants in the Navy had £5,000 a year, while the Army consultants had only the pay of their rank. Parliamentary action and newspaper comment have resulted in the termination of the engagements with the six medical men receiving £5,000 a year each as consultants at Royal naval hospitals. In the House of Commons on the 1st instant Dr. Macnamara announced that the new arrangement made with medical consultants at the Royal Naval hospitals was that seven consultants should be given temporary commissions as Surgeons-General, with the pay and allowance of that rank—namely, full pay £1,300 a year, house allowance £70—and two temporary commissions as Fleet surgeons of ten years' seniority, with the pay and allowances of that rank—namely, full pay £638 15s. a year, house allowance £50, and hospital allowance £53. As in the case of other naval medical officers, they had been informed that their appointments would not entirely preclude their engaging in private practice, provided it was clearly understood that such practice did not interfere with the performance of their naval duties.

### An Unexpected Result.

These announcements indicate an almost exactly opposite result to the profession to that which Sir William Osler advocated. He hoped that the Army consultants would at once be put on the same footing as their brethren in the Navy, though he intimated that the tone of the questions in Parliament suggested a feeling that the pay of their rank would be quite sufficient. Sir William sincerely hoped that this narrow view would not commend itself to Parliament; but, in fact, the narrow view seems to have triumphed. The medical profession as a whole has responded nobly to the "call of sacrifice in this crisis," and "in the most distant parts of the Empire professional men have given up large remunerative practices to join the Forces." Not only is it an injustice, but in the light of the payment of members of Parliament it is really monstrous for Parliament to entail heavy losses upon a group of men selected for their special abilities. "Many of them have fixed charges, domestic and educational, from three to four times in excess of the medical pay of a lieutenant-colonel in the Army. Some, of course, can afford the sacrifice, but others, though making it willingly, cannot do so without injustice to their families. An absence of a year or two puts a consultant out of touch with his *clientèle*; these men have to face not only an immediate but a prospective loss of income." In making these points clear Sir William Osler has rendered service to the profession, Parliament, and the public.

It will, therefore, be plain since Sir William Osler's letter appeared in the *Lancet* of the 26th ultimo, and as we have just shown, that the levelling down process has been carried out and less than justice has been done to war consultants. We welcome the discussion which has arisen, because it raises the whole question of the relation and position of all branches of the medical profession in this country at the present time. There is a general feeling in the profession that, as a rule, the fashionable rather than the competent make fortunes in medicine. The competent may be fashionable and often are, so it is reasonable to let the man who can make £5,000 to £10,000 a year in consulting practice stay at home and make it. No doubt a good many consultants may have passed the ten or fifteen years of "cakes and ale" of which Sir William Osler speaks, and see their incomes as consultants decreasing rather than increasing. As in the case of lawyers, so in the case of physicians and surgeons, it is only the relatively few who enjoy the "cakes and ale" attendant on great earning power.

Parliament and the advisers of the Government appear by their recent action to have come to the decision that a mistake was made at the outset in aiming at securing eminent consultants. The Government seem to have awoke to the fact that it is a common mistake to confuse competence with public recognition or fashionableness. There are many competent surgeons who for the most part are unknown outside the small circle of their immediate work and connection. As time advances some become fashionable and make money, others remain little known though they do excellent work in their sphere; some in general practice, some in the R.A.M.C. or the I.S.M. or other public services.

### The Young First-class Surgeon.

The men the Admiralty and War Office want, we believe, are the young men of high qualifications, thirty years of age or so—first-class surgeons who have not yet made a great name, and who are not probably making enormous incomes. Such surgeons are as good now as they ever will be. Many of them may be better—more competent, that is—than many of those more widely known. Such men would be as useful as surgeons as any members of the profession, and would not despise a colonel's pay. Possibly the Government have felt this to be true, and their present policy is to set about getting these men, who will do as good work as the others. Parliament and the public are credited with the feeling that it is a mistake for the State to pay the fees which over-rich individuals pay for fashion's sake. The new policy, if it is pursued, must result in leading the majority of the most fashionable and renowned consultants to continue their practice in the large centres of population at home.

### Alleged Jealousy and Difficult Cases.

The new policy would bring forward the men who are beginning to press up to and crowd the steps of the ladder which lead to fashionable reputation. Such men would be most welcome and helpful at the base hospitals at Boulogne, Havre, and wherever these are situated. Each one of them would be consulted for his operative skill, and his work should be to operate in cases of unusual difficulty, and to advise, when asked to do so, on cases of obscurity and doubt. A large number is not necessary. Most of the cases of wounds are within the competence of the R.A.M.C. and the civil surgeons enlisted for the war. Experience proves that the percentage of unusual difficulty is not great. Where difficult cases exist the R.A.M.C. men would be, as they have proved at Boulogne, only too thankful to be able at once to call for the services of a surgeon specially selected for his skill as an operator. We have satisfied ourselves it is not the fact, and it is unjust to suffer the impression, that jealousy would prevent the calling in of such surgeons when appointed as consultants. Surgery is a definite science, a handicraft in which a man knows, and must know, his limitations, and he is therefore glad of help in difficulties.

We have felt it desirable to state the actual need which exists for competent and experienced help at the base hospitals. We have thought it desirable also, in the interests of efficiency, to discourage the habit of running round in motor-cars sightseeing and gossiping in the casualty clearing stations, and at the headquarters of field-ambulances. All such proceedings should be promptly prevented and ended. The character of the work created by the great war at the bases and other hospitals, and generally throughout the ambulance system, demands that efficiency shall everywhere prevail not only throughout the R.A.M.C. organisation, which has proved so wonderfully efficient during the present war, but throughout the system by which consultants are supplied to co-operate with and to help the working staff of the hospitals in cases of unusual difficulty and urgency. We are confident that the modifications here indicated, and the change of system which they entail, whilst reducing the cost, are calculated to maintain the maximum of efficiency and to secure successful results without friction.

